# Learning from the implementation of a surgical opioid reduction initiative in an integrated health system: a qualitative study among providers and patients

**DOI:** 10.1186/s43058-024-00561-4

**Published:** 2024-03-11

**Authors:** Willemijn L. A. Schäfer, Julie K. Johnson, Meagan S. Ager, Cassandra B. Iroz, Reiping Huang, Salva N. Balbale, Jonah J. Stulberg

**Affiliations:** 1 Northwestern Quality Improvement, Research, & Education in Surgery (NQUIRES), Department of Surgery, Northwestern University Feinberg School of Medicine, 633 North Saint Clair Street, 20th Floor, Chicago, IL USA; 2 Mathematica, Chicago, IL USA; 3 American College of Surgeons, Chicago, IL USA; 4 Division of Gastroenterology and Hepatology, Department of Medicine, Northwestern University Feinberg School of Medicine, Chicago, IL USA; 5 Department of Surgery, University of Texas Health Science Center at Houston, Houston, TX USA

**Keywords:** Implementation evaluation, Contextual factors, Qualitative research, Acute care, Opioid reduction

## Abstract

**Background:**

Surgical opioid overprescribing can result in long-term use or misuse. Between July 2018 and March 2019, the multicomponent intervention, Minimizing Opioid Prescribing in Surgery (MOPiS) was implemented in the general surgery clinics of five hospitals and successfully reduced opioid prescribing. To date, various studies have shown a positive outcome of similar reduction initiatives. However, in addition to evaluating the impact on clinical outcomes, it is important to understand the implementation process of an intervention to extend sustainability of interventions and allow for dissemination of the intervention into other contexts. This study aims to evaluate the contextual factors impacting intervention implementation.

**Methods:**

We conducted a qualitative study with semi-structured interviews held with providers and patients of the general surgery clinics of five hospitals of a single health system between March and November of 2019. Interview questions focused on how contextual factors affected implementation of the intervention. We coded interview transcripts deductively, using the Consolidated Framework for Implementation Research (CFIR) to identify the relevant contextual factors. Content analyses were conducted using a constant comparative approach to identify overarching themes.

**Results:**

We interviewed 15 clinicians (e.g., surgeons, nurses), 1 quality representative, 1 scheduler, and 28 adult patients and identified 3 key themes. First, we found high variability in the responses of clinicians and patients to the intervention. There was a strong need for intervention components to be locally adaptable, particularly for the format and content of the patient and clinician education materials. Second, surgical pain management should be recognized as a team effort. We identified specific gaps in the engagement of team members, including nurses. We also found that the hierarchical relationships between surgical residents and attendings impacted implementation. Finally, we found that established patient and clinician views on opioid prescribing were an important facilitator to effective implementation.

**Conclusion:**

Successful implementation of a complex set of opioid reduction interventions in surgery requires locally adaptable elements of the intervention, a team-centric approach, and an understanding of patient and clinician views regarding changes being proposed.

Contributions to the literature

- To date, studies of opioid reduction initiatives in surgery have evaluated and demonstrated effectiveness on minimizing opioid prescriptions. In this study, we extend this evidence by investigating the implementation process of an opioid reduction intervention.
- We derived practical recommendations for initiatives from our evaluation using the Consolidated Framework for Implementation Research (CFIR).
- We found that opioid reduction initiatives in surgery require locally adaptable elements of the intervention, a team-centric approach, and an understanding of patient and clinician views regarding changes.
- These recommendations can be used for implementation of other similar initiatives.

## Background

Prescription opioids remain a driver for the “opioid epidemic” in the USA [1]. Opioids prescribed following surgeries significantly contribute to this epidemic and can result in long-term opioid use [2–4]. It is estimated that, annually, 5.7 million Americans continue to fill opioid prescriptions more than three months after their surgery, constituting 6.9% of patients undergoing surgery [5]. Long-term use is indicative of both chronic pain resulting from the procedure [6–14] as well as non-medical use [3]. Additionally, overprescribing of opioids after surgery impacts people within the larger social environment of the individual patient. On average, 70–90% of dispensed opioid pills remain unused after surgery [8, 9, 15–17]. For the 9.5 million annual nonmedical users of prescription opioids (approximately 2.9% of the U.S. population), unused medications obtained from friends and family was the most common source [18]. To prevent non-medical use of leftover opioids, it is crucial to avoid overprescribing in surgical settings.

Growing awareness of opioid overprescribing in surgery prompted numerous quality improvement initiatives across the USA [19–26]. Best practices to reduce the amount of opioids used in the perioperative setting, while maintaining adequate pain control, include prioritizing non-opioid analgesics [27, 28], setting pain management expectations, educating patients on the benefits and potential risks of various pain medications [12, 27, 29–31], and clinician education on alternatives to opioid analgesics for pain management [27].

**Fig. 1:**
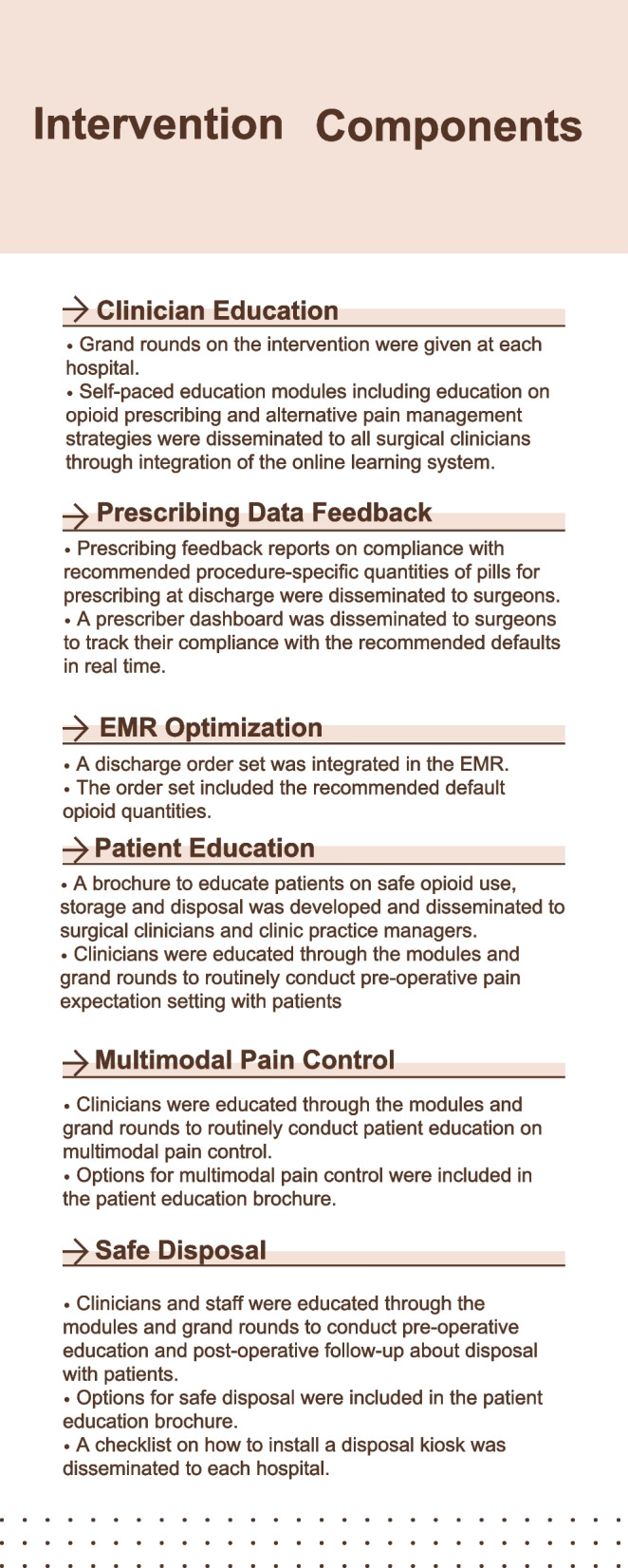
The intervention incorporates six components targeting clinicians and patients, including provider and patient education, prescribing feedback reports, electronic health record order sets with procedure-specific default opioid quantities, and opioid disposal

Based on national guidelines and evidence of best practices, we developed a multicomponent intervention, Minimizing Opioid Prescribing in Surgery (MOPiS) aiming to minimize opioids prescribed and used at and following discharge [32]. The intervention incorporates six components targeting clinicians and patients, including provider and patient education, prescribing feedback reports, electronic health record order sets with procedure-specific default opioid quantities, and opioid disposal (Fig. 1). The intervention was implemented in general surgery clinics across five hospitals throughout one health system between July 2018 and March 2019.

The MOPiS intervention successfully reduced opioids prescribed at discharge, and this was true even when adjusting for temporal trends [33]. Other studies of opioid reduction initiatives in surgery have evaluated and demonstrated effectiveness on minimizing opioid prescriptions [22, 34, 25]. Beyond clinical outcomes, it is important to understand the implementation process of an intervention. Such information can be used to extend sustainability of interventions and for dissemination of the intervention into other contexts [35, 36]. The implementation process can be evaluated by assessing how contextual factors impacted the success or failure of an intervention. Contextual factors are constructs that have been associated with effective implementation related to the intervention, outer setting, inner setting (hospitals and clinics), individuals (patients, clinicians, other stakeholders), and the implementation process [36].

In this study, we use qualitative methods to evaluate the contextual factors that affected implementation of the MOPiS intervention. Based on the evaluation, we aim to identify overarching themes and recommendations that can be used for implementation of other similar initiatives.

## Methods

### *Study design and sample*

We conducted a qualitative study to investigate stakeholder and patient experiences with the implementation of the multicomponent intervention. Data were collected in five general surgery clinics at five hospitals within the private Northwestern Medicine health system in Chicago, IL, between March and November of 2019. This includes one larger clinic within an inner urban area and four smaller clinics in suburban areas. Purposive sampling was employed to identify interviewees. Firstly, we selected all five general surgery clinics based on their participation in the MOPiS intervention. Secondly, within each clinic we invited the stakeholders (both clinicians and non-clinicians targeted by the intervention) of each clinic via phone calls and e-mails. Thirdly, in the waiting room of the clinics, the researchers also invited patients of the participating surgeons for face-to-face interviews following their consultations. All adult, English-speaking, patients who visited the general surgery clinic for a postoperative visit on the days of data collection were invited to participate in a one-time interview after their consultation. Interviewees in each clinic were invited until saturation was reached for the respective clinic.

The study was approved by the Northwestern University Institutional Review Board (STU00205053). To report study methods and results, we used the Consolidated criteria for reporting qualitative studies (COREQ) 32-item checklist.

### Data collection

A multidisciplinary research team including a PhD expert in qualitative research methods (JJ), an academic surgeon with a PhD and MD (JS), three PhD health services researchers (WS, SB, RH), and two Master’s level health services researchers (MA and CI), developed semi-structured interview guides (see Appendices 1–2). All researchers are female, except for JS who is male. The interview guides focused on the implementation of the six intervention components and explored how this was affected by contextual factors. Intervention components covered in each of the interview guides depended on the role of the interviewee. For example, patients were asked about the education that they received regarding pain medications, and clinicians were asked about their routine use of the educational brochures. Development of the questions on contextual factors was guided by the Consolidated Framework for Implementation Research (CFIR) [36]. The interview guide incorporated questions about the factors impacting the implementation process. For example, clinicians were asked about the barriers that they encountered in providing patients education about postsurgical pain management.

The interview guides were pilot tested in one clinic with two patients and two surgical residents and then further refined based on discussions with the multidisciplinary research team. The changes included shorter questions for patients and the removal of duplicate questions for all respondents. The interviews with the clinicians were conducted (by authors MA, JJ, WS) in person or via phone. The interviewees had no prior relations with the interviewers but were provided with information about the goal of the study and the role of the interviewer. All interviews with patients were conducted in person (by authors MA, JJ, WS). In some cases, a patient family member or friend was present during the patient interview. All interviews were audio-recorded upon consent of the interviewee, and we did not take field notes. The recordings were transcribed verbatim and not returned to the participants for comments. All transcribed interviews were de-identified upon completion, but participant roles were retained. Transcripts were not shared with the interviewees. Finally, all transcripts were transferred into MAXQDA software (Version 2018, VERBI Software GmbH, Germany), for coding and analyses.

### Coding and data analysis

Upon completion of the interviews, our research team developed a codebook covering the six MOPiS intervention components (patient education, clinician education, multimodal pain control, prescribing data feedback, EHR optimization, and safe drug disposal) and the constructs of the CFIR on contextual factors.

Researchers WS, JJ, and MA coded all transcripts in dyads, using a deductive logic, following the CFIR. Following independent coding, the coded transcripts were discussed by the dyad and any discrepancies were resolved with a third researcher.

Following the coding process, content analyses were conducted using a constant comparative approach. The coded data was discussed with all researchers to identify overarching themes within and across the intervention components of how the contextual factors contributed to implementation of opioid reduction initiatives. Data from the five surgical clinics and stakeholders (clinicians, staff, and patients) were analyzed concurrently to triangulate data. Results were not discussed with the interview participants.

## Results

We interviewed 45 participants, including 15 clinicians (8 nurses, 4 surgeons, 1 nurse educator, 1 advanced practice nurse (APN), 1 surgical resident), 1 quality lead, 1 surgery scheduler, and 28 patients. Patient interviews lasted approximately 15 min and clinician interviews lasted approximately 30 min. Of the staff, 4 interviewees were male and 13 were female. The number of participants varied between hospitals depending on the size and willingness of clinicians and patients to participate. Four clinicians (two attending and two resident surgeons) and eight patients declined to participate. The clinicians indicated that they did not have the time to participate, while the patients were not asked to provide a reason for their non-participation.

We identified three broad themes of needs related to conducting opioid reduction initiatives: [1] Reasons for variability in adopter responses to the intervention, [2] Surgical pain management as a team effort, and [3] Prior established patient and clinician beliefs regarding opioid risks as a facilitator. Sub-themes are denoted in bolded text.

### Theme 1: Reasons for variability in adopter responses to the intervention

We identified a high variability in responses to the intervention, rooted in various contextual factors including their individual characteristics, preferences, and adaptability of the various components. Table 1 includes representative quotes from this theme and its sub-themes.

**Table 1:** Representative quotes for the theme “Reasons for variability in adopter responses to the intervention”

Sub-themes	Intervention components	Representative quotes for the sub-theme and intervention component	Contextual factors related to this sub-theme (CFIR Constructs)
A. Variability in clinicians’ and patient’s characteristics and preferences	Prescribing data feedback	Nurse educator interviewee [about the prescribing feedback dashboard]: *Do we think that they're getting in there and looking?* Quality lead: *Dr. [name] I do know is looking. She has talked about several times like how it's driven her change. She has also looked at her nurse practitioners. Dr. [name] has looked a couple of times I believe…But, he has some ideas about quality sending him his reports. ..he wants us to [send the reports].*	Innovation characteristics (design quality and packaging)
	Clinician education modules	Surgeon: *Well, the computer modules, in all honesty, are kind of a pain. I spend way too much time on the computer doing things, so doing another thing, responding to another email, is just very time consuming, so it's actually more, probably, I don't want to say convenient, if it's in a form held at the surgery department meeting where someone has 15 to 20 minutes to talk about it. You got all the surgeons there, everybody can hear their questions*.	Innovation characteristics (design quality and packaging)
	Prescribing data feedback reports	Interviewer:..the final intervention was the surgical prescribing report and you said that you opened it.. Do you recall how you were doing or did you have any questions about it or any thoughts after seeing it? Surgeon 1: *I was doing very poorly. I was like at the very bottom. I was one of the worst people.. I was definitely more proactive after that about every time I work with a new resident going over the correct number for each operation was.* Surgeon 2: *Without a report card, I know that I'm prescribing less and my patients are getting less than they were two years ago. It's a huge difference.*	Characteristics of individuals (knowledge and beliefs about the intervention)
B. Need for interventions to be locally adaptable	Patient education: brochure	Patient interviewee: *I had to call Dr. [name]'s office the next day to remind myself of the flip flopping Tylenol and Ibuprofen protocol, because I think the way their software is set up for these papers, if you get the prescription, the printout only gives you the prescription information, but because I didn't want to take the prescription, I [crosstalk] remind myself of the protocol of flip flopping*	Innovation characteristics (design quality and packaging)

### *Sub-theme A:* Variability in clinicians’ and patient’s characteristics and preferences

Whereas some clinicians and staff responded favorably and quickly adopted the practices, others did not. For all components, except for multimodal pain management strategies, we found variability in the adoption of the component between clinicians. Variability in the responses, at least in part, stemmed from variability in clinicians’ characteristics and preferences for the “design” of the intervention components (CFIR constructs “Characteristics of individuals—Knowledge & beliefs about the intervention” and “Innovation characteristics—Design quality & packaging”). For instance, there was variation between prescribers in how they preferred to receive the feedback reports on their opioid prescribing data. Some surgeons preferred to receive individualized reports in their email, whereas others liked using the dashboards to review the prescribing within their practice. A nurse expressed the desire to share the reports publicly in the lounge to spur competition. Clinician preferences for “design quality and packaging” also varied for the education modules, e.g., during the interviews some clinicians indicated that they would have preferred in-person training to the virtual modules that were provided. The variability was also visible on the patient side, for example in whether they read the pain management information brochure.

### Sub-theme B: Need for interventions to be locally adaptable

The second sub-theme from the interviews with clinicians was that implementation could be impacted by the adaptability of the intervention to meet the needs of clinicians and of patients (CFIR construct “Innovation characteristics - Design quality & packaging”). The interviews revealed that some of the intervention components were not adaptable to the local needs of clinicians and patients, whereas others were. The brochures including patient education on safe opioid use were created for the hospital system and could not easily be adapted or edited by each clinic, as any changes to the format or content would require additional review and approval from the system’s patient education department. Further, several clinicians indicated that they would have preferred to integrate the information from the brochure within existing materials and others noted that the materials were only available in English. The lack of adaptability was also visible in the experiences of patients, e.g., one patient indicated that they needed to call their doctor about how to take non-opioid medications, as this information was not included in the standardized brochure.

### Theme 2: Surgical pain management as a team effort

Second, we identified that surgical pain management involves a broad team and implementation success varied when not all stakeholders were engaged and the relationship between the various team members are not considered. Table 2 includes examples and representative quotes of this theme and the sub-themes.

**Table 2:** Representative quotes for the theme “surgical pain management as a team effort”

Sub-themes	Intervention components	Representative quotes for the sub-theme and intervention component	Contextual factors related to this sub-theme (CFIR constructs)
A. Need for engagement	Clinician education	Interviewer 2: *As best you can remember, has there been anything that's come through about opioid prescribing?* Nurse interviewee: *No.* Interviewer 2: *There hasn't been a meeting or anything and people have talked about extending to do this?* Nurse interviewee: *Nope.* Interviewer 2: *Would that be helpful to you?* Nurse interviewee: *Mm-hmm (affirmative). Well and I think part of it is, it would be helpful for the follow-up portion when patients are calling and asking for refills.*	Process (engaging)
	Prescribing data feedback	Interviewer: *And then as part of the program we have also sent out reports to the individual prescribers about your prescribing habits. Were you aware of these reports?* Nurse interviewee: *No, they don't really share that with us.*	Process (engaging)
	Clinician education: modules	Interviewer: *And do you also remember if what you learned from [the education modules]?* Nurse interviewee: *Yeah it did because you know, we educate before surgery and so we are now mentioning that and preparing patients for that when we do our education prior to surgery.. It gives you some background so it is not something that where I specifically have a in depth conversation with the patient but if the patient is asking questions about pain meds or something, for example, we used to say you will go home with pain meds, Well I don't say that anymore because they may not go home with pain meds.* .	Process (engaging)
B. Hierarchal relations between care providers	EMR optimization (order sets)	Resident interviewee: *And sometimes there are attendings who are very old school and always prescribe the same amount every time, and they'll tell you "Give them 15 of this", even though the order says "Oh, you should only be giving five." And as a resident, you can't go against the attending who's saying that, so then we'll be noncompliant, and then it would be like well is that really their fault. So I don't know. But I think now, they did send that email where we can look up the thing.*	Inner setting (culture)

### Sub-theme A: Need for engagement of all stakeholders

The first sub-theme, need for engagement of all stakeholders (CFIR construct “Process—Engaging”), highlighted that people from specific roles, including nurses and non-clinical professionals, were sometimes overlooked in the implementation of specific components. A surgeon indicated that disseminating the prescribing data feedback was important for residents as well as the primary surgeon as the residents are the ones responsible for most of the opioid prescribing. Initially, the feedback reports were only shared with the primary surgeons, but this was extended to other prescribers including residents based on feedback. This modification to include more of the team members helped making more team members active participants in the intervention. Except in a few instances, nurses were not aware of the prescribing data feedback reports, and this lack of team integration likely limited project success for those teams. When nurses were aware of the reports, they often ensured the reports were reviewed at regular meetings.

Regarding clinician education, while many surgeons attended grand rounds where the opioid initiative was discussed, nurses, as well as other professionals, did not attend these presentations. A nurse indicated that being informed about the grand round lectures would have been helpful as this would have been important information in their role of responding to patient phone calls discussing refills after discharge. A surgery scheduler also indicated that being more informed would have helped them reinforce patient education (e.g., surrounding the importance of disposal). The clinician education modules, on the other hand, were disseminated to all clinicians involved in the surgical care pathway. This supported their goal of minimizing opioids, not just by prescribing fewer but also through nurses tailoring their patient education, e.g., a nurse indicated that she learned from the module that she should no longer tell patients that they go home with pain medications.

### Sub-theme B: Hierarchical relationships between care providers

We also identified hierarchical relationships between care providers that could interfere with adoption of certain intervention components (CFIR construct “Inner setting—Culture”). Resident surgeons were exposed to the education modules and prescribing tools, but they voiced concerns about prescribing in ways that were not aligned with what the attending surgeon requested. Discharge pain medication prescribing after inpatient stays is often managed by surgical residents, yet the ultimate responsibility for the patient’s well-being lies with their supervising attending surgeon. Therefore, while the order sets provided residents with a tool to align their prescribing habits with the health system recommendations, there was sometimes tension if the attending surgeon habitually prescribed a higher number of opioids at discharge than what was recommended for specific procedures by the health system. Residents expressed that they felt pushed into an uncomfortable position by this tension. While clinicians were aware of the order sets, they were not universally adopted. A resident indicated that they felt supported by an email with information about the order sets and quantities providing them something tangible to back-up their choice in their communication with attending surgeons.

### Theme 3: Prior established patient and clinician beliefs regarding opioid risks as a facilitator

Finally, we saw that the established awareness of the risks associated with opioids contributed to the success of intervention implementation (CFIR construct “Characteristics of individuals-Knowledge & beliefs about the innovation”). Table 3 includes representative quotes from this theme. Both patients and clinicians referenced already knowing about many risks of opioids and reasons to avoid or limit their use. When asked, patients consistently indicated knowing about risks such as addiction and side-effects. Various factors impacted patients’ knowledge, including what they had heard in the media, negative experiences of friends who became addicted, and their own experiences with opioids from prior procedures. As a result, patients were receptive to the patient education and their providers’ plan to minimize opioid prescribing. For example, they consciously followed their doctor’s instructions referencing long term risks of opioid use in the form of heroin addiction. Patients appeared to less frequently fill their prescriptions when they did not consider the opioids necessary to manage pain. Receptiveness to the change was also visible in patients’ expressions of satisfaction with pain management. Ultimately, most patients indicated that they considered their pain manageable, in some cases with opioid use and in some cases with alternative pain management strategies alone, such as acetaminophen and ice packs.

**Table 3:** Representative quotes for the theme “Prior established patient and clinician beliefs regarding opioid risks as a facilitator”

Sub-themes	Intervention components	Representative quotes *for the sub-theme* and intervention component	Contextual factors related to this sub-theme (CFIR constructs)
Awareness of opioid risks	NA	Patient interviewee: *“I know I come from the inner city here in [city], so I know a lot about opioid addiction. I've got friends that had problems when I was growing up with heroin. I've had recently a couple of friends, I work in an industry, the food service industry. I used to bounce bars, I used roadie for bands, so I know a lot of band members that have OD'd and are no longer with us.”*	Characteristics of individuals\knowledge and beliefs about the innovation
	Patient education	Patient interviewee: *“I was like, I manage my pain. I'm good.”* Interviewer: *“And you took the medications was discussed with your doctor?”* Patient: “*Completely. Yeah, I've used it before. I mean, I've had a few surgeries. I know about pain management, I know how to take that stuff, like I said, I'm very stubborn. Set in my ways, where it's just like, you know what? It hurts, but you know what? It doesn't hurt any worse than if I were to get addicted to heroin*.”	Characteristics of individuals\knowledge and beliefs about the innovation

The clinicians also indicated that they were already aware of many risks associated with opioids and, in some cases, practices focused on minimization of opioids were already incorporated in their workflow. For example, because of implementation of Enhanced Recovery Protocols for some procedures, patients already received education on minimized opioid use. Therefore, it was clear that the media attention to this topic and general understanding of opioid risks were helpful in improving implementation.

## Discussion

Many surgical procedures result in enough pain to require some opioids to manage the pain. However, there is a need to balance the benefits of the opioids prescribed with the associated risks. In the USA, reducing surgical prescribing is more important than ever given that the opioid epidemic continues to worsen in recent years [1] and physicians continue to prescribe high amounts of opioids after surgery [37]. The epidemic is not isolated to the USA either, with increases in opioid-related deaths observed in, for example, England, Sweden, and Lithuania [38].

This study identifies three key themes from the implementation evaluation of a complex, multicomponent intervention to reduce the quantity of opioid pills prescribed at surgical discharge in a diverse health system. First, we found high variability in the responses of clinicians and patients to the intervention, highlighting the necessity for locally adaptable components, especially in patient and clinician education materials. Second, surgical pain management should be recognized as a team effort. We identified specific gaps in the engagement of team members and found that the hierarchical relationships between surgical residents and attendings impacted implementation. Third, we found that established patient and clinician views on opioid prescribing were an important facilitator to effective implementation. From the identified themes, we can draw several practical recommendations. While some of these recommendations, such as the importance of adaptability, are applicable to and well-known from implementation of interventions in other contexts, some recommendations specifically relate to opioid reduction initiatives, such as the importance of a multi-component approach, and to the surgical context, for example the importance of considering hierarchy.

The first recommendation is that it is essential to make intervention components adaptable to address individual patient and provider needs and the hospital-specific context. This was underlined by the variability of their responses to each intervention component. Stakeholders were consulted prior to implementation of the MOPiS intervention, and we identified specific needs for the intervention design which supported implementation [19]. Even though the intervention components aligned with the clinician preferences in many cases, there were needs for further adaptations. For example, the patient education brochures did not always contain the information that providers wanted to have included and could not be adapted. Previous studies have shown that adaptability can increase feasibility and acceptability of an intervention. Simultaneously, it can have the unintended consequence of lowered fidelity to implementation and a subsequently diminished effect on outcomes [39–42]. Therefore, practitioners need to receive guidance on adaptations [41–43]. Further, there may be boundaries, legal and within health system policies, to the possibilities of making interventions adaptable. For the MOPiS intervention, such boundaries were encountered, e.g., from a legal perspective, opioid disposal boxes could only be placed in a specific place in the hospital where security can be guaranteed. Additionally, it is important to consider the balance between adaptability of elements and fidelity to the intervention [44].

Second, even if the intervention components are adaptable to individual needs, there is still a need for a multi-component strategy to address opioid prescribing behavior because some individuals may not be receptive to specific components, which we observed for the individual feedback reports. The intervention employed multiple strategies to change clinicians’ opioid prescribing behavior, for example by providing them with individualized feedback on their prescription behavior and by providing them an online education module. An extensive review of techniques to change physicians’ behaviors showed that there is not a unifying approach that is effective and therefore multiple interventions yield better results [45]. Our data further supports this assertion.

The third recommendation was the need to engage all team members across the surgical continuum, including surgeons, all nurses (e.g., clinic, pre-op, post-op, floor), residents, and nonclinical staff such as schedulers. Within the MOPiS implementation process, the importance of engagement of some team members was sometimes overlooked. While grand rounds presentations were successful at engaging prescribers, the opportunity to engage people in other roles through this mechanism was missed. To ensure the success of each intervention component, there is a need to recognize that surgical pain management is a team effort. Although surgeons in many cases are the prescribers of the actual medications, other behaviors can support or act as a barrier to the desired change in prescribing. For example, while the feedback reports were designed for and disseminated to prescribers, other staff might have been able to support the use of these reports, also including pharmacists. Prior to implementation, it is important to ensure understanding of which staff members may encounter the patient and how they are involved in pain management when targeting surgical opioid reduction in a specific setting. This can be done, for example, through patient journey mapping which maps the steps of patients through their “care journey” including interactions with health professionals [46]. Based on that information, tailored education to all clinicians and staff can avoid inconsistent messaging towards patients.

The fourth recommendation was that behavior change interventions in surgery need to account for the role that the hierarchal relationships within the field play and how it impacts behavior. This is particularly relevant to opioid prescribing as it relates to a surgical residents’ role and attending surgeon beliefs and practices. This hierarchy should be considered in the implementation process of the intervention. A systematic review identified “negative hierarchy” hampering quality improvement and resulting in anxiety and fear [47]. Empowerment of people at all levels of care to advocate for safer care practices around opioids can potentially be supported by tools such as checklists on team compliance with protocols [47, 48]. In our initiative, the order set, in combination with an email communication, empowered residents to prescribe according to the new recommendations. However, as the hierarchical relationships are persistent and may not be fully overcome by empowerment tools, there will remain a need to change the beliefs of the attending physicians first.

Finally, we identified that the current culture surrounding opioid use in medicine presents us with an opportunity for change. In the specific context of the USA, both clinicians and patients are acutely aware of the risks of opioids. As a result, patients are willing to limit their opioids following surgery and this creates an environment for successfully implementing prescribing reduction initiatives. Similar to our study, another qualitative study among surgical patients revealed widespread awareness among patients about opioid medications which informed their intentions about using opioids [49]. Ultimately, the patients within our study reported that they were satisfied with their pain management. Likewise, a systematic review on behavioral interventions to decrease opioid prescribing after surgery found that of 18 studies the majority of studies did not find worse pain control following reduced prescribing initiatives [50]. In addition, a statewide opioid reduction effort also found that, following implementation of default quantities at discharge, despite significant decreases in both prescribing and consumption, patient-reported satisfaction and pain scores remained stable [26].

Our assessment prior to the MOPiS implementation showed that some physicians were worried about a negative impact on patient satisfaction rates [19]. Such concerns may cause resistance to changing prescribing practices. The accumulated knowledge about impact on patients can be used when introducing similar interventions in other populations to convince clinicians that changing prescribing behavior does not lead to lower patient satisfaction.

### Limitations

There are several limitations of this study. Some stakeholder groups are underrepresented in our study, including non-clinical staff and non-English speaking patients, meaning that some perspectives were missed. Nevertheless, by involving a wide variety of stakeholder groups including patients and various clinician groups, we were able to triangulate perspectives and identify themes universally considered to be important. Second, the study did not focus specifically on refined implementation outcomes, such as reach and sustainment but rather on implementation broadly as experienced by the staff and patients. Specific implementation outcomes are hard to measure reliably using only qualitative data and would require a mixed-methods approach including other resources such as electronic health record data. Third, the sample of clinicians who participated in the interviews is small and may represent a more engaged group who are interested in reducing opioid prescriptions. However, the sample represents a large portion of the prescribers who were exposed to the intervention.

### Implications

We identified several recommendations focusing on the need for adaptability of intervention content, engagement of and communication with stakeholders encountering the patients in their entire surgical journey, and importance of considering developments in the outer setting context. These lessons can be considered by other health system representatives when initiating a similar initiative. Future research should more directly measure how strongly these implementation themes relate to the effectiveness of the intervention.

## Conclusions

Successful implementation of a complex opioid reduction intervention in surgery requires using multiple strategies simultaneously for behavior change and knowledge acquisition. Adaptations should be allowed, and the implementation should be team-centric across the surgical continuum.

## Data Availability

Not applicable.

## Appendix 1

### Patient Interview Guide Questions

1. Can you start by telling me what you know about the problem with over-prescribing opioids for patients?
  a (If aware of opioid epidemic) Can you tell me what you’ve heard about this issue? From whom? (e.g., TV, friends, family)

Now I’d like to ask you about the education you received around your (most recent) surgery.

2. What do you remember about the education you received before your surgery (in your pre-op visit(s))?
  a Who provided the education before your surgery (e.g., surgeon, nurse)?
  b Who talked to you about your pain medication?
  c Did your provider(s) give you brochures/ handout / other materials? If yes, did you read those materials?

3. Can you tell me what your provider(s) told you before your surgery (in your pre-op visit(s)) and after your surgery (at discharge) about:
  - [*For all items clarify at what point(s) this information was provided and if messaging was consistent*]
  - a. How to manage your pain at home?
  - b. Safe use of pain medications?
  - c. Safe storage of pain medications?
  - d. Disposal of pain medications?
  - e. Other pain management therapies to try at home (e.g., Tylenol, ice, massage)?

4. Have you been able to understand all the information you’ve been given related to pain management? Have you received answers to all your questions (if any)?

Next, I’d like to ask you a few questions about your expectations around pain management.

5. Can you tell me what your providers told you about what to expect in terms of pain after your surgery?
6. What were your expectations about pain management before surgery? During your stay in the hospital? During your recovery after discharge?
7. Since your surgery, have your providers helped you set expectations and milestones around pain management (e.g., expectations for when you should be able to run errands comfortably, exercise, return to all your usual activities)?
  a Can you tell me what they told you? Who has talked with you about this (e.g., surgeon, resident, APP, nurse, pharmacist)

8. Have the milestones or expectations been realistic to achieve for you? Why or why not?
  a Has your level of pain been manageable?
  b What are you doing to manage your pain? Are you taking any medications? If yes, what kind? (e.g., Norco, Tylenol) What about any other pain management therapies? (e.g., ice, massage)
  c What has been most effective?

**Conclusion: **Is there anything else that you would like to tell us about, or make sure that we learn about while we are here today?

## Appendix 2

### Clinician Interview Guide Questions

1. Let’s start by talking about prescribing opioids to treat post-surgical pain at [hospital]. Prior to the summer of 2018, did [hospital] have any existing policies, procedures, or guidelines around prescribing opioids, post-surgery? If yes, how about at the department level?
2. [NAME] was here to present about the project on [DATE]. Were you able to attend his presentation? What steps were taken by the hospital/your department before and following this presentation?
3. What, if anything, has shaped the way you prescribe opioids for surgical patients? What about the pain management education you provide to patients?

Now, I’d like to discuss your experiences with the opioid reduction efforts.

4. **First I want to talk about the provider education component of the intervention.**
  - a) Are you aware of the opioid educational modules released in November 2018?
  - a. If yes: Have you completed the educational module?
  - i. If no: Why not?
  - b. How has it changed your practice?
  - i. Probe: One topic addressed in the modules was pain management expectation setting with patients. In what ways, if any, have your conversations with patients about pain management expectations changed due to the module?
  - ii. Probe: The module also addressed non-opioid pain management alternatives. In what ways, if any, have your recommendations for multimodal, non-opioid pain control changed due to the module?
  - iii. Probe: Additionally, the module discussed best practices for talking with patients and families about opioid safety. In what ways, if any, have your conversations with patients about opioid safety changed due to the module?
  - iv. If module hasn’t changed practice: Why not?
  - b) Have you ever participated in any other formal education around post-surgical opioid prescribing? (If yes) Can you tell me more about that?

5. **The second component of our intervention is patient education around post-surgical pain management and opioid safety. We have developed standardized patient information brochures.** *[Show brochure]*
  - *[For residents, APPs, and hospitalists, the following questions refer to patient education post-op/at discharge]*
  - a. Have you seen the educational brochure that we developed?
  - i. If yes: How are you using the brochure? In what ways, if any, has the use of the brochure changed your practice? If not using: Can you explain why not?
  - b. What other education, if any, do you provide patients about post-surgical pain management strategies? And specifically about opioid safety?
  - c.What are the barriers you encounter in providing education to patients about these topics?
  - d. Who documents in Epic that education was provided to the patient? Where in the chart is this documented and how?

6. **The third component of our intervention is to decrease opioid prescribing so that patients receive prescription quantities in line with current recommendations.**
  - a. Who prescribes opioid medications to [hospital] patients at discharge? Surgeon/ resident/ PA/ NP?
  - b. How do you determine the quantity of pills to prescribe?
  - c. Do you generally use discharge order sets when prescribing? Do your residents/other prescribers?
  - We launched updates in Epic to lower default opioid prescription quantities in discharge order sets and to include pain management groupings in late August. *[Show print-out of recommendations and tip sheet]*
  - d. Are you aware of the updated opioid prescription quantity defaults and pain management groupings in Epic discharge order sets? How did you find out about them?
  - i. If yes: Are you using the default quantities in the discharge order set/ do you tell your residents to use them for your patients?
    1. If yes, using defaults: How has this affected your practice?
  - 2. If no, not using: Why not? What are the barriers involved?
  - e. What are your thoughts on the pain management order groupings?
  - f. Due to the Epic updates, has there been any change in the extent to which you recommend multi-modal non-opioid pain management strategies while patients are in the hospital? At discharge? Please elaborate.

7. **Fourth, we have developed and sent out reports with surgical prescribers’ individual prescribing data.** *[Show example of report if needed]*
  - a. Are you familiar with these reports?
  - b. Have you opened this report? Why/why not?
  - i. To what extent are the reports easy to interpret? Were your results surprising?
  - ii. How has seeing the report data changed your practice, if at all?

8. **Thinking about these four components, how do you think the intervention could change post-surgical opioid prescribing at your hospital?**
  a What are the barriers to further implementing each of the four components? (e.g., infrastructure, resources)
  b Can you tell me how your patients have responded to changes due to the intervention? Have you elicited information from patients regarding their experiences with post-surgical pain management after changing your practice? If so, what have you heard from patients?

9. **Finally, I’d like to ask you about activities to increase safe disposal of unused opioid pills at your hospital.**
  a Were you aware of the National Prescription Drug Take Back Day on October 27^th^, 2018?
  b Were you aware that [your hospital] participated in the October Take Back Day?
  c What guidance, if any, do you give patients about safe disposal of their unused opioids?

**Conclusion: **Is there anything else that you would like to share with us?
